# Enjoying the Free Menu? Discoursing the Barriers to Exclusive Breastfeeding for Improved Maternal and Child Health in Tanzania: A Review of Evidence

**DOI:** 10.1002/hsr2.71905

**Published:** 2026-02-28

**Authors:** Chakupewa Joseph Mpambije

**Affiliations:** ^1^ Department of History Political Science & Development Studies; Mkwawa University College of Education (MUCE): A Constituent College of the University of Dar es Salaam Iringa Tanzania

**Keywords:** exclusive breastfeeding, maternal and child health, Tanzania

## Abstract

**Background:**

Despite the emphasised benefits of exclusive breastfeeding (EBF) among pregnant and breastfeeding mothers being recognised globally, its practice is still low among developing countries, including Tanzania. Several barriers to EBF in Tanzania have been detailed but lack a comprehensive approach. This study provides comprehensive evidence of the barriers to EBF, drawing from existing studies in Tanzania.

**Methods:**

This review was conducted using search queries in several databases, including PubMed, Cochrane, Scopus and Web of Science. The review included qualitative, quantitative and mixed methods studies written in English addressing EBF in Tanzania. The critical appraisal skills programme checklist was used to assess the quality of studies while thematic analysis was used to analyse the data for each identified study.

**Result:**

Twenty‐three studies (23) from 10 regions met inclusion criteria, including eight qualitative studies, nine cross‐sectional studies, four mixed methods approaches, and two quantitative studies. All the studies reported maternal, infant and social‐environmental to EBF practices in Tanzania.

**Conclusion:**

Though this review has shown evidence of barriers to EBF, further research is needed, especially on factors influencing EBF practices in Tanzania.

## 1 Introduction

Maternal and child health (MCH) has remained a global concern. The emphases on reducing maternal mortality rate (MMR) and infant mortality rate (IMR) are enshrined in the sustainable development goal (SDG 3) [1]. The World Health Organisation (WHO) recommends that each country attain MMR and IMR of 70 deaths per 100,000 and 12 deaths per 1000 live births by 2030, respectively [1, 2]. Globally, MMR and IMR have significantly decreased by over 40% and 30%, respectively, from the 2000s to 2020; however, most developing countries have exhibited unsatisfactory progress [2, 3]. Despite the remarkable effort to reduce MMR and IMR incidences, the 2020 data indicate that MMR and IMR have remained higher worldwide, standing at 223 maternal deaths per 100,000 live births and 37 infant deaths per 1000 live births, which is below the recommendations of the WHO and international standards [2, 4]. The root causes of the deaths include factors associated with pregnancy complications during and after delivery, childbirth, and the postpartum period [4]. While least developed countries are said to represent 95% of all occurring maternal deaths, sub‐Saharan Africa (SSA) represents about three‐quarters of all global maternal deaths [2]. In particular, the average MMR and IMR across SSA have remained higher, at 531 deaths per 100,000 live births and 27 deaths per 1000 live births, respectively [2, 5].

Unlike developing countries, developed countries have attained the pace towards meeting WHO recommendations, whereby more than 120 countries worldwide have achieved SDG‐3 [4]. As for developing countries, interventions towards improving MCH, MMR, and IMR have been implemented, especially in SSA, emphasising adherence to exclusive breastfeeding [6, 7, 8]. For example, the WHO recommends exclusively breastfeeding for 6 months following the introduction of complementary foods [9]. This means mothers should solely breastfeed their infants within 6 months, and continue normal breastfeeding for 2 years [9]. The WHO target is to attain 50% of the EBF prevalence rate by 2025 [9, 10]. However, the global EBF prevalence rate is still low, standing at 38%, which is 12% below the WHO target [10]. Data shows that when optimised, EBF may prevent 0.82 million child deaths, which represents about 12% of all child deaths in resource‐constrained countries [11]. Moreover, breastfeeding protects a child from non‐communicable diseases and lowers the risk of mortality and infections of diseases, including obesity, diabetes, and asthma [11]. Again, breastfeeding has been associated with improved maternal health as it helps to strengthen the uterus after delivery, and is sometimes linked with preventive measures, including preventing maternal breast and ovary cancers [6]. Moreover, evidence shows that stunting among 150,000,000,000 children due to chronic malnutrition may have been prevented through EBF [12]. Despite the recognised importance of EBF over child growth and development, the EBF prevalence rate has remained low, especially in developing countries [13]. For instance, in South Africa, although about 90% of infants are breastfed shortly after birth, only 32% remain exclusively breastfed at 6 months of age [14]. Similarly, the EBF prevalence rate in Ethiopia has remained low for decades, at 60% [15].

Tanzania has also experienced low EBF prevalence below the WHO recommendations, decreasing from 53% since the 2000s to 45% in 2020 [16]. Furthermore, data indicated that only 45% of infants aged 4–5 months were still exclusively breastfed by their parents [17]. Despite the significant role played by EBF practices to improve MCH [6, 8, 11], breastfeeding mothers encounter diverse hurdles towards fulfilling the WHO recommendations [8, 18]. Studies show that EBF among breastfeeding mothers suffers from maternal, infant and social‐environmental factors. As regards maternal‐related factors, Mogre [19] found that a lack of knowledge about EBF has affected most mothers' tendency to exclusively breastfeed their infants. Again, a lack of encouragement and support from spouses and family has been mentioned as affecting EBF practices [20]. Furthermore, factors such as insufficient milk production, nipple pain, HIV‐positive status, and maternal age significantly increase the likelihood of not exclusively breastfeeding [21]. Thus, exclusive breastfeeding is influenced by multiple factors. Infant characteristics, such as illness and persistent crying, can pose challenges to maintaining EBF. Maternal circumstances, including employment, and insufficient support from husbands or relatives, as well as environmental challenges such as unsafe working conditions and the need to breastfeed in public, further affect breastfeeding practices [18, 22].

Based on the existing evidence, many studies have explored EBF practices in Tanzania, but there is no scoping review addressing the topic. This gap warrants this study in Tanzania, which comprehensively captures EBF field‐related studies conducted in different regions of Tanzania. The review also accentuates regions in Tanzania where more studies pertaining to EBF have been conducted, thereby pressing the need for conducting field‐based studies in those Regions to capture the most emerging issues limiting EBF. This study potentially informs policymakers and implementers on the factors limiting EBF in Tanzania to thoroughly design evidence‐based interventions for effective EBF practices. This will, in turn, contribute to improved MCH.

## 2 Methodology

### 2.1 Study Protocol and Registration

**Figure 1:**
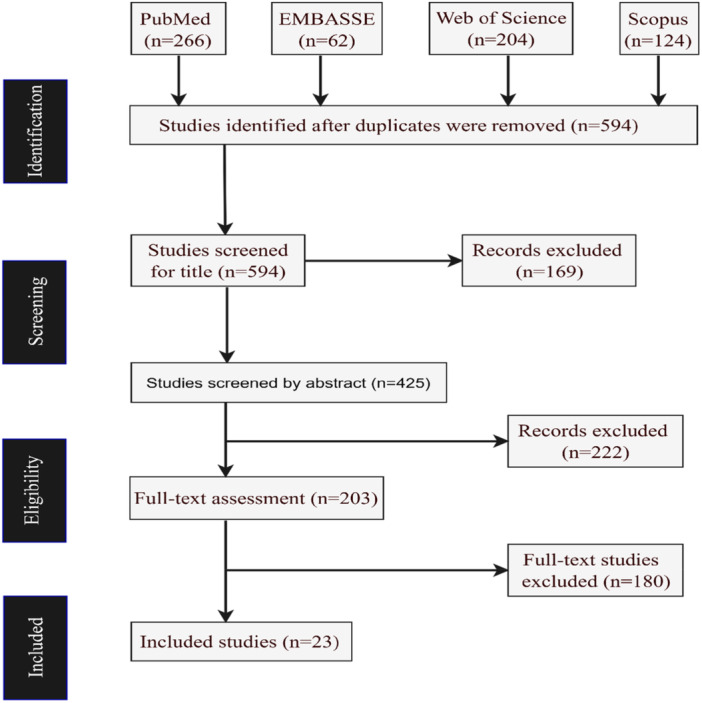
**A PRISMA flow chart**.

This scoping review was designed according to the guidance provided by the Preferred Reporting Items for Systematic Reviews and Meta‐Analyses (PRISMA) [23] (see Figure 1 for the PRISMA flow diagram). Thus, the objective of the current scoping review was to map the existing evidence on barriers to EBF practices in Tanzania. The author developed a study protocol but it was not registered through PROSPERO.

### 2.2 Search Strategy, Data Source and Eligibility Criteria

A comprehensive literature search was conducted across PubMed, Cochrane Library, Scopus, EMBASE, and Web of Science to identify English‐language peer‐reviewed studies published between 2015 and 2025 on exclusive breastfeeding (EBF) in Tanzania. The review included both quantitative and qualitative studies presenting empirical data from Tanzania while excluding commentaries, editorials, reviews and studies from other countries. The search strategy used free‐text terms, tailored to each database. The specific strings for each data base were as follows: PubMed: (“exclusive breastfeeding” OR “breast feeding”) AND (“barriers” OR “challenges” OR “determinants” OR “hindrances”) AND (“Tanzania” OR “Zanzibar”), restricted to English language from 2015 to 2025; Scopus: TITLE‐ABS‐KEY(“exclusive breastfeeding” OR “breast feeding”) AND TITLE‐ABS‐KEY(“barriers” OR “challenges” OR “determinants” OR “hindrances”) AND TITLE‐ABS‐KEY(“Tanzania” OR “Zanzibar”), restricted to articles in English from 2015 to 2025; EMBASE: (‘exclusive breastfeeding’ OR ‘breast feeding’) AND (“barriers” OR “challenges” OR “determinants” OR “hindrances”) AND (“Tanzania” OR “Zanzibar”), and Web of Science: TS = (“exclusive breastfeeding” OR “breast feeding”) AND TS = (“barriers” OR “challenges” OR “determinants” OR “hindrances”) AND TS = (“Tanzania” OR “Zanzibar”), in English from 2015 to 2025. All retrieved records were compiled, and duplicates were removed before screening to ensure a thorough and systematic identification of relevant primary research.

### 2.3 Identification and Selection of Studies

All identified studies were imported into Microsoft Excel, and duplicate studies were eliminated. After reviewing the title and abstract of each identified study, the author screened the studies independently to assess their eligibility, followed by a full‐text assessment. This assessment was evaluated based on the study objectives, methodology, population, and research findings. Studies that did not meet the inclusion criteria were removed for further analysis.

### 2.4 Thematic Analysis

The findings from the 23 studies were analysed using a thematic analysis approach. Key findings related to barriers to EBF were initially extracted independently by the author and a second reviewer to ensure accuracy and minimise bias. Each finding was coded line by line, using qualitative data management tools as appropriate. Similar codes were grouped into categories, which were then organised into overarching themes reflecting common barriers across the studies. Any discrepancies in the theme were resolved through discussion between the author and the second reviewer to ensure consistency. This process allowed for the identification of patterns and relationships among maternal, infant, household, and environmental factors affecting EBF in Tanzania.

### 2.5 Quality Assessment

Studies that were selected for retrieval in the second phase were assessed independently by the author. The Critical Appraisal Skills Programmes (CASP), version 2018, was used to determine the quality of each included qualitative, quantitative and/or mixed method study [24]. The tool has two main features: a section for assessing the bias of the implementation outcomes, and sections for describing the implementation strategy and intervention. Studies with a 50% or above were included because they often had essential information for this study.

### 2.6 Data Extraction

Studies were independently imported into the Microsoft Excel spreadsheet for data extraction by the author. Study characteristics were extracted, including the author's first name, study design and setting, methodology, publication date, study objectives and associated barriers to EBF to improve MCH.

## 3 Results

The database search yielded 656 records for analysis, of which 594 were available for screening. Based on study criteria, 203 full‐text studies were assessed for eligibility, of which 23 were eligible for inclusion (Figure 1). Another 23 studies, representing unique studies for this review, were included in the final evaluation (Table 1). These studies included [11, 25, 26, 27, 28, 29, 30, 31, 32, 33, 34, 35, 36, 37, 38, 39, 40, 41, 42, 43, 44, 45, 46].

**Table 1:** Characteristics of the included studies.

Authors name	Title	Study objective	Study participants and sample size	Study design & Setting	Findings
[25] Kazaura, M. 2016	Exclusive breastfeeding practices in the Coast Region, Tanzania.	To examine EBF practices in rural settings of Coast Region, Tanzania	342 mothers of reproductive age	A cross‐sectional (Coast region)	Exclusive breastfeeding is affected by insufficient maternal milk, employment, and traditional customs.
[26] Rasheed et al. 2018	Adherence to Exclusive Breastfeeding and Associated Factors in Mothers of HIV‐Exposed Infants Receiving Care at Kilimanjaro Christian Medical Centre, Tanzania	To determine factors that influence adherence to EBF in mothers of HIV‐exposed infants receiving care at Kilimanjaro Christian Medical Centre.	128 mothers with HIV‐exposed infants aged 6 to 12 months	A cross‐sectional (Kilimanjaro)	Barriers to EBF practices among many women included maternal insufficient milk and employment.
[27] Mahande et al. 2015	Factors Affecting Exclusive Breastfeeding among Women in Muheza District Tanga Northeastern Tanzania:A Mixed.	To examine factors that affect EBF practice among women in Muheza district, Tanga Region, Tanzania.	316 women with infants aged 6–12 months	A cross‐sectional (Tanga)	Insufficient milk, maternal demographic features, postpartum stress, and sick and crying babies lead to low EBF prevalence among women.
[28] Grounds S. 2021	Views of Breastfeeding in Public among Informally‐Working Mothers of Infants under 6 Months in Moshi Urban District, Kilimanjaro Region, Tanzania: A Qualitative Study	To understand the perceptions and views of breastfeeding in public among mothers of infants less than 6 months of age who work in the informal sector in Kilimanjaro Region, Tanzania.	20 women of reproductive age and 21 infants	A qualitative (Kilimanjaro)	Factors associated with low EBF practices among women were social‐environmental factors, including maternal employment and breastfeeding in public places.
[29] Mgongo et al. 2019	Facilitators and Barriers to Breastfeeding and Exclusive Breastfeeding in Kilimanjaro Region, Tanzania: A Qualitative Study	To explore social and cultural factors that influence the practice of EBF in Kilimanjaro region.	78 women who were still breastfeeding and with infants aged 0–12 months	A qualitative (Kilimanjaro)	A component that affects most mothers from EBF includes breastfeeding in public areas, which can be explained through fear of the evil eye and witchcraft beliefs—maternal demographic characteristics including maternal age, breast engorgement, crying infant and breastfeeding in public places.
[30] Augustino et al. 2024	Barriers to exclusive breastfeeding practice among HIV‐positive mothers in Tanzania. An exploratory qualitative study	To explore individual, household, and community obstacles in the Ilala district, Dar es Salaam.	HIV‐positive breastfeeding mothers, health facility‐based healthcare providers (clinicians), and Community Health workers.	An explanatory qualitative (Dar es salaam)	Insufficient milk, maternal demographic characteristics, breast‐associated challenges and maternal employment were the main hindrances to EBF practices.
[31] Matare et al. 2019	Barriers and Opportunities for Improved Exclusive Breastfeeding Practices in Tanzania: Household Trials with Mothers and Fathers	To explore barriers and facilitators to EBF in rural Tanzania and assess parents' Willingness and ability to try specific recommended EBF practices plus strategies for men to support breastfeeding.	36 mothers and 30 fathers with infants aged 0 and 5 months	Households trials/qualitative (Lake zone)	Traditional beliefs include insufficient milk and maternal employment as the constraining factors to EBF practices among women.
[32] Augustino et al. 2023	Infant and Young Child Feeding in the Context of HIV: An Exploration of barriers in Exclusive Breastfeeding practice in Dar Es Salaam, Tanzania.	To explore individual, household, and community Obstacles in the Ilala District, Dar es Salaam.	HIV‐positive breastfeeding mothers, healthcare providers, and Community Health workers	A qualitative study (Dar es Salaam)	The barrier to EBF practices among many women: conditions such as insufficient milk, postpartum stress, and maternal occupation have affected EBF prevalence.
[11] Maonga et al. 2016	Factors affecting exclusive breastfeeding among Women in Muheza District Tanga Northeastern Tanzania: A Mixed Method Community‐Based Study.	To examine factors that affect EBF practice among women in Muheza district, Tanga region, Tanzania.	326 women with infants aged 6–12 months	A mixed method (Tanga)	Maternal demographic characteristics, including low education about EBF practices, insufficient milk, and sick and/or crying babies, were obstacles to the EBF practices and prevalence of most women.
[33] Mgongo et al. 2018	“We give water or porridge, but we don't really know what the child wants:” A qualitative study on women's perceptions and practices regarding exclusive breastfeeding in Kilimanjaro region, Tanzania	To explore the knowledge, attitudes and practices of EBF among mothers in Kilimanjaro region of northern Tanzania.	78 women with infants aged 0–12 months who were still breastfeeding	A qualitative (Kilimanjaro)	Barriers reported include insufficient milk, crying babies, traditional beliefs, and health‐related factors such as advice from health professionals, which were termed common barriers that deter EBF practices.
[34] Hashim et al. 2017	Predictors of appropriate breastfeeding knowledge among pregnant women in Moshi Urban, Tanzania: a cross‐sectional study	To assess the understanding of women on optimal breastfeeding during pregnancy and the performance of the health system in reaching women with information on EBF	536 pregnant women who were in their third trimester and attending routine care.	A cross‐sectional (Kilimanjaro)	As shown statistical significance, common obstacles include maternal demographic characteristics, including marital status, age, and education.
[35] Dede et al. 2020	Exclusive breastfeeding patterns in Tanzania: Do individual, household, or community factors matter?	To examine the combined individual, household, and community implications in explaining exclusive breastfeeding patterns in Tanzania.	998 mothers with infants	A cross‐sectional (Tanzania)	Perceived infant‐maternal hindrances to EBF practices among many mothers were age and weight of the infant and maternal age. Also lack of husband support and support from nurses.
[36] Craig et al. 2023	“Because of mchango, I give my baby gripe water so he sleeps and stops crying”: Exclusive breastfeeding and parents' concerns about colic‐like symptoms in infants under 6 months in Lake Zone, Tanzania.	To explore parents' use of non‐prescribed medicine in response to infants' colic‐like symptoms during the EBF period.	36 mothers of infants 0–5 months and 30 men	A qualitative (Lake zone)	Perceived illness, non‐prescribed medicine such as traditional medicine, and crying babies affected exclusive breastfeeding among many mothers.
[37] Eliufoo et al. 2024	Exclusive breastfeeding experience among healthcare working mothers in central Tanzania: A qualitative study	To explore healthcare‐working women's experiences with EBF	15 female health workers	A qualitative study (Dodoma)	Challenges while maintaining and balancing EBF with work, such as lack of time, consideration of alternative milk as a result of insufficient milk, and physiological discomforts. Significantly, these prevented most working mothers from fully practising EBF.
[38] Mwantimwa. 2018	Demographic determinants of access to and usage of breastfeeding information among parents in Mbeya City, Tanzania	To examine the influence of demographic characteristics on access to and use of breastfeeding information among parents in Mbeya City, Tanzania	70 parents with children aged between 0 and 2 months	A quantitative (Mbeya)	Demographic characteristics such as unmarried mothers, low level of education, age, and low income were barriers for most women to EBF practices.
[39] Kokushubira et al. 2017	Factors affecting exclusive breastfeeding among Postnatal Mothers in Kinondoni Municipality, Dar es Salaam	To assess mothers' perceptions of EBF, complementary food given to children, and factors that affect breastfeeding.	100 mothers who had breastfed their babies for 6 months	A cross‐sectional (Dar es salaam)	Busy with chores without a helper, crying baby, crackled nipples, sick mother, insufficient milk and lack of education affected mothers' EBF prevalence.
[40] Jahanpour et al. 2022	Role of clusters in exclusive breastfeeding practices in Tanzania: A secondary analysis study using demographic and health survey data (2015/2016)	To estimate the determinants of exclusive breastfeeding	13,000 women aged 15–49 and 3200 men aged 15–49	A mixed method (Tanzania)	The barriers to EBF practices among mothers with infants aged between 0 to 6 months included maternal demographic features such as age, and education and infants' demographic characteristics such as age and weight.
[41] Mchome et al. 2020	“When I Breastfeed, It Feels as if my Soul Leaves the Body”: Maternal Capabilities for Healthy Child Growth in Rural South‐eastern Tanzania	To identify maternal capabilities for ensuring healthy child growth	Key informants include community health workers (CHWs) and Traditional Birth Attendants (TBAs). Sample size specifically not mentioned	A mixed approach (Morogoro Region)	Insufficient milk, maternal demographic features, postpartum depression, women's employment and traditional beliefs and values affected most mothers to fully implement EBF in their infants.
[42] De Bruyn et al. 2017	Characterising infant and young child feeding practices and the consumption of poultry products in rural Tanzania: A mixed methods approach	To measure the impact of agricultural interventions on the diets and growth of children	503 children from eight rural villages in Singida region	Mixed method (Singida Region)	Early cessation of breastfeeding and introduction of complementary foods were associated with insufficient milk among many mothers having infants aged between 0 to 6 months.
[43] Dede et al. 2019	Determinants of infants feeding practices in Tanzania: a cross‐sectional analysis among breastfeeding mothers in Masasi district, Tanzania.	Assess the determinants of infants' mixed‐feeding practices by identifying the common supplements introduced to infants before 6 months of age.	Mothers/women of reproductive age of 15–49 years with infants under the age of six months	A cross‐sectional (Mtwara region)	Infant age and weight were barriers that prevented women from feeding their infants exclusively.
[44] Lyellu et al. 2020	Prevalence and factors associated with early initiation of breastfeeding among women in Moshi Municipal, northern Tanzania.	To determine the prevalence and factors associated with early breastfeeding initiation among women in northern Tanzania.	328 pregnant women	Quantitative (Kilimanjaro Region)	Mode of delivery, such as women who give birth by virginal delivery, had low EBF prevalence rates.
[45] Jahanpour et al. 2023	Mapping regional variability of exclusive breastfeeding and its determinants at different infant ages in Tanzania	To investigate regional disparities in EBF practices and identify determinants of EBF among infants aged 0–1, 2–3 and 4–5 months in Tanzania.	Women and men of reproductive age	A cross‐sectional (Tanzania)	Maternal social‐demographic characteristics, infant age, and maternal employment affect EBF practices.
[46] Mandara et al. 2024	The relationship between feeding practices and stunting among children under 2 years in Tanzania Mainland: A mixed‐method approach.	To assess the relationship between feeding practices, stunting, and barriers among children under 2 years of age.	Infants of 6–23 months age group	A cross‐sectional (Tanzania)	Factors associated with low EBF prevalence rates among mothers with children of 6 months and below were maternal demographic characteristics, maternal employment, and infant demographic characteristics.

### 3.1 Study Characteristics

The present study employed studies conducted between 2015 and 2025 on barriers to EBF in 11 regions of Tanzania. These included Coast region (4.4%), Dar es Salaam (13%), Dodoma (4.4%), Kilimanjaro (26.1%), Mbeya (4.4%), Morogoro (4.4%), and Mtwara (4.4%). Others were Singida (4.4%), Tanga (8.7%), and Lake Zone (8.7%); while 17.4% of the studies were conducted in multiple regions. Of all the 23 included studies, there were 8 (34.8%) qualitative studies, 9 (39.1%) cross‐sectional studies, 4 (17.4%) mixed methods studies and 2 (8.7%) quantitative studies, as detailed in Table 1. The included studies identified barriers to EBF practices, including maternal factors such as insufficient milk [11, 25, 26, 27, 29, 30, 31, 32, 33, 34, 37, 38, 42], maternal demographic characteristics [11, 29, 30, 34, 38, 40, 41, 46], breast‐related factors [29, 30, 37, 39, 42], and postpartum stress [27, 32, 41]. Twelve studies [11, 27, 29, 33, 35, 36, 37, 39, 40, 43, 46] described infant‐related factors, and 10 studies described social‐environmental factors deterring EBF practices [11, 25, 26, 28, 29, 30, 31, 32, 33, 37, 39, 41, 46]. The majority of the included studies posed insufficient maternal milk and maternal employment as significantly deterring EBF practices in Tanzania (Table 3). Several quotes from qualitative studies were included from eight studies [11, 28, 29, 31, 32, 33, 36, 37] as illustrated in Table 2.

**Table 2:** Selected quotes exploring the barriers to EBF practices in Tanzania.

Citation	Theme/sub‐themes	Selected quote
[11] Maonga et al. 2016	Insufficient milk	*I gave him water and soft foods when he was three months old and when he was five months old, I gave him thick porridge. He was given these foods early because of insufficient milk* (Mother‐in‐law 1, aged 58). [Pg.6].
[29] Mgongo et al. 2019	Maternal demographic features	*“In our community, young girls like me (aged 18‐ 25 years) do not like to breastfeed. They are afraid that their breasts will sag and their body shapes can change”* [Pg.4].
[32] Augustino et al. 2023	Postpartum stress/depression	*I have been suffering a lot from pregnancy with no support, and now I must raise this child alone. Because I have no support, I have to feed my child complementary foods so that he may stay for along lime without milk* [Pg.8‐9].
[29] Mgongo et al. 2019	Breast‐associated factors	*“The child burped on my breast and I experienced breast pain. I went to the hospital, and I found I had a cracked nipple. The nurse at the health clinic advised me to give porridge.”* [Pg.5].
[36] Craig et al. 2023	Crying infant	*The difficult part can be when the child cries due to mchango, and he refuses to breastfeed. The last option is to give him gripe water/any prescribed medicines. –21‐year‐old mother of 1‐month‐old son* [Pg.53].
[37] Eliufoo et al. 2024	Maternal employment	*“I breastfed my baby every 2 h while on maternity leave, but it was difficult once I started working again. I breastfed both before and after work to acclimate, but occasionally, I felt like my milk supply had reduced”* [Pg.266].

**Table 3:** Matrix of reviewed papers addressing maternal, infant, social‐environmental and health system barriers to EBF practices in Tanzania.

Reference	Insufficient milk	Breast and health factors	Postpartum stress	Maternal demographic features	Sick and crying infant	Infant age and sex	Maternal employment	Breasts in public places	Traditional beliefs and norms
[25] Kazaura 2016	√						√		√
[26] Rasheed et al. 2018	√						√		
[27] Mahande et al. 2015	√		√		√				
[28] Grounds 2021							√	√	
[29] Mgongo et al. 2019		√		√	√	√		√	
[30] Augustino et al. 2024	√	√		√			√		
[31] Matare et al. 2019	√						√		√
[32] Augustino et al. 2023	√		√				√		
[11] Maonga et al. 2016	√				√				√
[33] Mgongo et al. 2018	√				√				√
[34] Hashim et al. 2017				√					
[35] Dede et al. 2020						√			
[36] Craig et al. 2023					√				
[37] Eliufoo et al. 2024	√	√			√		√		
[38] Mwantimwa 2018				√					
[39] Kokushubira et al. 2017		√			√				√
[40] Jahanpou et al. 2022				√		√			
[41] Mchome et al. 2020	√		√	√			√		√
[42] De Bruyn et al. 2017	√	√			√				
[43] Dede et al. 2019						√			
[44] Lyellu et al. 2020			√			√			
[45] Jahanpour et al. 2023		√	√						
[46] Mandara et al. 2024				√		√	√		

### 3.2 Barriers Associated to Maternal Factors

Based on thematic analysis, maternal‐associated factors were identified as the main barriers to EBF prevalence among mothers in Tanzania. This theme emerged from five sub‐themes, namely insufficient milk, maternal demographic characteristics and EBF practices, postpartum stress, maternal breast‐associated challenges and mode of delivery.

#### 3.2.1 Insufficient Milk

Most of the included studies documented insufficient milk as a common barrier to EBF practices in Tanzania. Data indicated that the low quantity and quality of maternal milk affected EBF among many breastfeeding mothers [11, 32, 33, 37, 41, 42]. Low production of maternal milk was attributed to food insecurity, poor nutrition and inaccessibility of nutritious meals [30, 33, 37]. In most cases, very few mothers could afford three meals per day. As a result, they produce small quantities of milk that are not enough to feed their infants exclusively [30], and light milk that requires breastfeeding an infant frequently for satisfaction [37]. Four studies found that insufficient milk among mothers made them unable to exclusively breastfeed their children [25, 26, 27, 41]. For example, one respondent in Maonga's study supported this view, thus;

I gave her water early, and she started eating light porridge at three months. When she was about five months old, we made the porridge a bit stiff, made from millet. Of course, we did this because breast milk was not enough and was too light so that she could get a little bit. (Mother‐in‐law 1, aged 58).

[11, Pg.6]

Similarly, Matare [31] found that insufficient milk among mothers has forced male partners to buy other foods that a baby requires, including giving them porridge, a situation that underscores the cessation of EBF. The situation has increased discomfort among many employed mothers who work for many hours [37].

#### 3.2.2 Maternal Demographic Characteristics and EBF Practices

Among the commonly cited risk factors are maternal demographic features that affect EBF practices among feeding mothers, including maternal knowledge, age, and marital status [29, 30, 34, 38, 40, 41, 46]. Maternal age seems to strongly correlate with low EBF among mothers, especially younger mothers who prefer infant feeding for less than 6 months when they introduce light food [30, 46]. Despite the emphasised importance of EBF practices towards improved MCH, this knowledge has not permeated well among younger mothers [38]. This is especially true for younger mothers who live alone without any experienced mother, as they are less likely to breastfeed exclusively due to a lack of external forces to enforce EBF practices. For example, a study by Mchome [41] found that many young mothers avoid EBF, believing that it will tarnish their good appearance, a situation that subsequently leads to low EBF prevalence rates. Moreover, diversity among younger mothers' perceptions demonstrates that EBF can cause breast sag. As a result, many young girls stop breastfeeding to preserve their appearance in most communities [29, 41]. For instance, in a study by Jahanpour [40], EBF practices were higher among women aged 18 and above, and low among women below 18. Again, it was revealed that women aged below 24 years were more reluctant to practise EBF than mothers aged 24 and above [30]. Similarly, studies have found that knowledge about appropriate breastfeeding was three times higher among mothers aged 25–49 [29, 34]. As one participant revealed during FGD:

“In our community, young girls like me (aged 18‐ 25 years) do not like to breastfeed. They are afraid that their breasts will sag and their body shape will change. My friends asked me: Why are you breastfeeding? My child is four months now” FGD 8.

[29, Pg.4]

Again, marital status affects EBF practices and prevalence among many mothers. Studies found that most unmarried mothers lack support from their partners such as material support and food, unlike married mothers who normally have such support [38, 46]. Similarly, mothers with lower education levels are less likely to access and practice EBF information than educated mothers, who can access and practice the information [38, 46].

#### 3.2.3 Postpartum Stress

Several studies have highlighted factors associated with postpartum stress as deterring most mothers from EBF practices. Specifically, one study has indicated that women who rely on their own for food and other essentials significantly experience depression which makes them reluctant to practise EBF [32]. Also, women who lack support from their husbands experience postpartum stress [41]. This is because lack of support also leads to food insecurity, which is associated with reduced milk production among many mothers [32, 41]. During interviews, one participant attested that:

I have been suffering a lot from pregnancy with no support, and now I must raise this child alone. I know the effect of alternative feeds, but I don't believe it is my fault alone. If I cannot produce enough milk, there is no way I will leave him hungry with no food (P16, Health Centre).

[32, Pg 8‐9]

Moreover, food insecurity has been mentioned as a critical factor that has pushed many mothers from EBF [32]. In a study by Mahande, for example, participants had the perception that babies require other food nutrients, thereby affecting their EBF [27].

#### 3.2.4 Breast‐Associated Challenges

The barriers pooled from four studies [29, 30, 37, 39] that reported factors that deterred EBF practices among mothers showed that maternal health and condition, including HIV‐positive status, nipple pain and engorgement associated with breastfeeding, lowered the EBF prevalence rates. Studies have also found that burped breast is associated with early cessation of EBF among mothers [29, 39]. Burped nipples may be caused by a lack of experience associated with the attachment and positioning of child while breastfeeding among many mothers, which results in severe nipple pain [39]. A mother with burp breasts is not allowed to breastfeed until she gets medical treatment due to several reasons, including transmission of diseases [29]. As one participant narrated during FGD;

“The child burped on my breast, and I experienced breast pain. To cure this, I had to put the breast on my child's head, but it was not cured. I went to the hospital, and I found I had a cracked nipple. The nurse at the health clinic advised me to give the child porridge. My child was only 3 months” FGD 2.

[29, Pg 5]

Furthermore, breast sores and abscesses affect EBF among many mothers, especially those who are HIV positive [30, 39]. These sores and abscesses make mothers feed their infants using one breast, a situation that most mothers do not prefer since it results in breasts having different sizes and lengths [37, 39].

#### 3.2.5 Mode of Delivery

It was also found that the mode of delivery, whether virginal or caesarean, affects EBF among many mothers. Data indicate that mothers who give birth through the vagina are more likely to stop breastfeeding their infants. This is unlike mothers who gave birth by caesarean mode, who are less likely to stop breastfeeding their infants and start feeding their infants simple complementary foods after 6 months [44]. This is because mothers who give birth by caesarean mode have enough time to take care of their infants since they cannot perform some jobs for some months [45]. It is also reported that a woman who conceived while her infant was still young cannot proceed with EBF, knowing that it may harm the baby in the womb [41].

### 3.3 Infant‐Associated Barriers

The findings of the reviewed studies have shown that infant‐related factors can hinder EBF practices among many breastfeeding mothers. Study findings assert that low EBF prevalence in the country is also linked with certain conditions of an infant, including crying and sickness.

#### 3.3.1 Sickness and Crying

Multiple studies also found a connection between sick and crying babies and exclusive breastfeeding among mothers in Tanzania [27, 36, 37, 39]. Specifically, lower prevalence rates of EBF practices were associated with sickness and crying [39]. Crying babies were found to influence early cessation, and subsequent introduction of light foods and medicines, including gripe water [27]. In a study across the Lake Zone, participants posed gripe water as a non‐prescribed medicine for a crying baby [36]. Gripe water is also given to a crying child so that they can sleep; while some studies also mentioned the perception that gripe water can quench thirst in babies [36, 37, 39]. It is clearly stated that an infant's first breastfeeding is associated with several difficulties that require non‐prescribed medicine, including gripe water and some traditional medicines [29, 36]. This was supported by a study that found that diseases affected many mothers and their infants, including *Mchango. Mchango* is a disease that affects infants shortly after being breastfed by their mothers, and is associated with abdominal pain and makes a baby cry at night [29]. As one participant attested:

“My child was crying, and my friend told me that the baby had mchango. I was advised to send the child to the elders and do the abdominal cuttings and the child was also given medicine to lick. Then he got healed” FGD 1.

[29, Pg. 5]

Also, sick babies and those crying due to insufficient maternal milk were frequently cited to affect EBF [11]. It was evidenced that crying babies tend to refuse breast milk, and this makes mothers find alternative ways to feed their infants [33]. Baby refusing breast milk was a stigma to EBF among mothers as they feared that the child was not satisfied by the milk, and so they decided to give infants other foods before 6 months as recommended worldwide [11, 36].

#### 3.3.2 Age and Weight of the Infant

The infant's age has become a factor that influences low EBF among mothers [40, 43]. Data indicates that within two and above months, an infant starts eating more, and breast milk is not sufficient. As a result, a mother looks for alternatives, including giving an infant light food like porridge [43]. For example, in a study by Mgongo [29], it was highlighted that infants aged between 2 and 3 months [AOR = 0.5; 95% CI: 0.3–0.8] and 4–5 months [AOR = 0.1; 95% CI: 0.1–0.2] were less likely to have been breastfed by their mothers than infants aged 0–1 month, who breastfed frequently after delivery. Similarly, another study found that infants aged between 2 and 3 months were associated with about 60% (AOR0.35, 95% CI 0.23, 0.53) reduction in the adjusted odds ratio of EBF compared to infants aged < 2 months [35]. It was further found that infants born with a weight above average were less likely to have been breastfed exclusively than infants born with an average weight [35]. Similarly, the sex of an infant affects EBF practices; studies have found that female infants are breastfed more than male infants [46]. Table 2 addresses important selected quotes from themes and subthemes.

### 3.4 Social‐Environmental Factors

Studies have indicated that social‐environmental factors are another barrier to EBF prevalence among mothers. This theme emerged from three sub‐themes: maternal employment, traditional beliefs and norms, and breastfeeding in public places as hindrances to EBF practices.

#### 3.4.1 Maternal Employment

In this study, maternal employment was represented by 39.1% of all included studies that address factors that hinder EBF practices in Tanzania. Maternal employment, formal or informal, influenced many mothers to stop or rarely breastfeed their infants [25, 26, 28, 30, 32, 37, 41, 46]. In particular, mothers in formal employment are less likely to exclusively breastfeed their infants than mothers in informal employment. It was evidenced that in most cases, mothers in formal employment lack payment for maternity leave and, as a result, they do not stay long before returning to work to gain money [28, 30]. Also, employed mothers have little time to feed their infants, as they spend much time at workplaces where they leave their kids with housemaids or guardians to feed the infants on bottled milk [30, 37]. This explanation underscores the early initiation of soft food and cessation of EBF among many employed mothers, as one participant revealed:

“I breastfed my baby every two hours while on maternity leave, but it was difficult once I started working again. I breastfed both before and after work to acclimate, but occasionally, I felt like my milk supply had reduced”.

Participant 2 [37, Pg. 266]

It was also found that employed women lack time to breastfeed their infants upon their return to work [37]. This is because institutions do not offer time to breastfeed, a condition that makes employed mothers unable to balance between institutional needs and childcare. Since their work is important, most of them decide to look for alternative ways of feeding their infants, including formula milk and, sometimes, complementary foods [37]. On the other hand, self‐employed mothers prioritise farming activities, affecting EBF [46]. In most cases, mothers go early to farming activities, and sometimes lack lunch, while they need to breastfeed their infants. Because the mother has never taken food during the day, the milk becomes insufficient, and both starve [41]. Again, the working environment affects EBF among mothers as those who depend on themselves to search for food and money to meet family needs find it difficult to adhere to EBF [32, 38].

#### 3.4.2 Traditional Beliefs and Norms

Traditional norms, values, and beliefs are also highlighted as risk factors for EBF practices [39]. Data portray that the use of traditional medicines to cure infants and families is associated with low EBF in most rural areas [39, 41]. Similarly, studies posed the traditional tendency of mothers who used to give infants gripe water or medicine after they realised some symptoms of illness. For instance, a survey by Matare [31] revealed that communities tend to give infants medicine or gripe water to cure *mchango*, a disease associated with excessive crying. This situation has influenced early cessation of EBF and initiation of soft food in an infant [11, 31]. As evidenced by one participant:

I give my baby gripe water because of mchango. When I give it gripe water, the baby sleeps and stops crying.

[31, pg.6]

Furthermore, the influence of family members, including husbands, mothers‐in‐law and other relatives, has affected EBF among many mothers in Tanzania. Family members who have given birth do offer advice to breastfeeding mothers, referring to their experience in breastfeeding [25]. For instance, a study by Mgongo [33] highlighted factors associated with family advice that affect EBF. In a study by Kazaura [25], participants highlighted the influence of family members as they may stop exclusive breastfeeding and start complementary feeding.

#### 3.4.3 Breastfeeding in Public Places

Some of the included studies [28, 29] highlighted breastfeeding in public places as a factor that contributes to low EBF prevalence in Tanzania. Studies indicated that most mothers have negative perceptions regarding breastfeeding in public areas [28]. This is linked with such factors as shyness, inherited behaviour from their parents, the presence of unfamiliar people, unhealthy air, and evil eyes. Also, the fear that the infant may be bewitched by people with evil eyes has affected EBF in public areas [29]. The concept of the evil eye has made many mothers not breastfeed their infants in public places. As a result, most mothers opt for bicarbonates and, sometimes, charcoal, soil and eyebrow pencils to protect their infants from evil eyes [28]. One participant stated as follows during FGDs:

“Another thing we are advised when we leave home is to draw a child with an eyebrow pencil. They said it helps to protect a child from a person with an evil eye. We have been told eyebrow pencil helps like that…yes is like that I heard eyebrow pencil helps very much yes” (22‐year‐old mother of a 3‐month‐old)

[28, Pg.17]

In addition, breastfeeding in public places has been associated with the concept of ‘Zongo’ [28, 29]. The term ‘Zongo’ is traditionally understood to describe a condition affecting infants that is attributed to malevolent forces. Specifically, ‘eye Zongo’ has been interpreted as witchcraft, the ‘evil eye,’ or a form of bewitchment believed to compromise breastfeeding, spoil breast milk, or cause illness in a child following an attack by a mature woman [28]. Moreover, breastfeeding in public is often regarded as socially unconventional; many mothers report rarely observing older women engaging in public breastfeeding [28].

## 4 Discussion

Globally, exclusive breastfeeding has been emphasised over time. Specifically, the WHO recommends exclusive breastfeeding for 6 months after birth without any complementary foods. EBF practices have been mentioned to have a positive association with MCH and the reduction of child morbidity and maternal‐infant mortalities. However, despite the importance of EBF, its prevalence rate among mothers has remained low in Tanzania, standing at 45% in 2020 [16]. This indicates that almost 55% of infants in Tanzania are not exclusively breastfed for 6 months. Peer‐referred studies conducted in different regions of Tanzania pointed to common hindrances to EBF practices among mothers. In this review, common barriers that emerged among pregnant and breastfeeding mothers included maternal employment, insufficiency of maternal milk, crying and sickness of infants. Peer‐referred studies also revealed that readiness to practice EBF is affected much by the maternal working environment and lack of education about EBF among mothers, especially younger mothers. Also, factors associated with maternal illness, like nipple pain, infant age, and mode of delivery, have negatively impacted EBF practices.

Infant‐related factors, particularly frequent crying, emerged as a prominent barrier to EBF in multiple studies, being reported in eight (34.8%) of them [27, 36, 37, 39]. Crying infants often prompt mothers to introduce light foods or formula prematurely, thereby undermining adherence to EBF [27, 39]. Factors contributing to frequent crying, as reported in specific study contexts, include perceived insufficient milk supply, hunger, thirst, and illness [47]. In some cases, caregivers administer non‐prescribed medications in attempts to soothe the baby [48], which may further compromise breastfeeding practices [49]. Cultural beliefs attribute persistent crying to the “evil eye” [49], which was observed to reinforce early supplementation in the studied communities. While these findings provide valuable insights, however, they are context‐specific and cannot be generalised across all populations in Tanzania. Nevertheless, these barriers have broader implications for MCH in which infant crying can disrupt optimal feeding practices and increase maternal stress, thus affecting caregiving behaviours and well‐being [48, 49]. To address this challenge, multi‐level interventions tailored to local contexts are warranted, including community education programmes to demystify harmful cultural beliefs, training mothers in responsive breastfeeding techniques, and ensuring access to skilled lactation support to manage perceived low milk supply [50].

Maternal employment significantly emerged as a barrier to EBF, with 39.1% of studies reporting a negative impact of women's work on EBF [26, 30, 31, 32, 37, 41, 46]. These findings are consistent with evidence from other settings, including Indonesia, where working mothers were more likely to practice non‐EBF compared to non‐working mothers [51]. Indeed, formally employed mothers are faced with limited time for breastfeeding, as they are provided with brief maternity leave, frequently less than 2 months, forcing them to return to work shortly after childbirth [49, 50, 51, 52]. Similarly, self‐employed mothers face challenges in adhering to EBF recommendations due to labour‐intensive activities such as farming [52, 53, 54]. Insufficient milk supply further compounds these challenges, with studies reporting that mothers producing low milk volumes often fail to breastfeed exclusively, whereas those with adequate supply could comply if not disrupted by other barriers [37, 52, 55]. Insufficient milk was also associated with infant problems, including frequent crying due to hunger, which could prompt early introduction of light foods [49, 55, 56]. Misconceptions regarding breast milk, like the belief that it lacks sufficient water or is inadequate to relieve infant constipation, contributed to early EBF cessation [50, 52, 53, 54, 57]. Additionally, formula milk advertising may indirectly influence these practices, particularly for working mothers who perceive formula as a convenient solution when returning to work, although this influence may vary across communities [58]. The findings from this study while carrying important insights to EBF, still differ across contexts, specifically in Tanzania. The findings call for the need of workplace policies to extend maternity leave, regulating of formula marketing, and community‐based education programmes to address misconceptions about breast milk and support mothers in achieving EBF.

Among the included studies, five (21.7%) reported that maternal age and marital status influence EBF practices [29, 35, 40, 43, 46]. Mothers aged more than 24 years were more likely to practice EBF than younger mothers. This is consistent with evidence from Myanmar, where younger mothers were less likely to adhere to EBF guidelines due to perceived physical changes [56]. Common perceptions among younger mothers in the studied communities include beliefs that breastfeeding causes breast sagging, deformation of chest shape, or weight loss, which may lead to early premature introduction of light foods [49, 50, 53, 56, 59]. Similarly, in South Africa, young mothers reported feeling uncomfortable breastfeeding in public, which may hinder EBF practices [49]. Marital status also demonstrated an association with EBF, with married mothers more likely to breastfeed exclusively than unmarried mothers [47]. This was attributed to the financial and emotional support provided by partners, which enables mothers to focus on infant care. Implication from the findings suggests that the importance of implementing interventions targeting EBF promotion should consider the unique challenges faced by younger and unmarried mothers, including body image concerns, social stigma, and limited support. Strategies could include community education campaigns to normalise breastfeeding, peer support programmes, and policies aimed at guiding young mothers by enhancing adherence to recommended EBF.

While this scoping review provides a comprehensive overview of the barriers to exclusive breastfeeding (EBF) in Tanzania, the approach inherently produces context‐specific and qualitative insights, which may not be universally generalizable across all communities. Some factors, such as the influence of formula milk advertising or workplace‐related challenges, may vary by settings and were not always explicitly reported in the included studies. Consequently, the review is limited in its ability to assess the causal influence of specific barriers. Future research could build on these findings through more focused scoping reviews targeting particular subpopulations, such as working mothers, young mothers, or mothers with low economic status, or through primary empirical studies that quantify the prevalence and impact of specific barriers. Additionally, intervention studies evaluating strategies to overcome identified barriers would provide actionable evidence to inform policy and programmatic decisions on EBF.

## 5 Conclusion

In Tanzania, there are few primary and empirical studies exploring the status of EBF prevalence and associated factors. So, this study aimed to explore the common maternal, child and social‐environmental barriers to EBF in Tanzania. The results of this study show that the overall rate of EBF prevalence among many breastfeeding mothers was poor (45%); thus, the study can be interpreted with little caution due to the low variability of the studies. EBF practices are recommended to ensure MCH. Specifically, the WHO recommends that an infant be exclusively breastfed for 6 months, followed by complementary foods for up to 2 years. The common barriers to EBF practices in Tanzania were found to be insufficient milk, maternal employment, mode of delivery, maternal health, and baby crying. This calls for policymakers and programme initiators to make efforts towards sensitising the community, mostly women, to breastfeed their infants exclusively. This should include developing interventions to promote EBF practices among breastfeeding women to strengthen their awareness of the importance of EBF practices.

The study found that most women have an understanding of the importance of EBF, but most breastfeeding women do not fully practice EBF. To increase EBF prevalence rates, interventions should focus on strategies to minimise the cited hindrances. Health workers should also mobilise women on the importance of EBF and incorporate mobilisation programmes during antenatal and postnatal visits and counselling. Regarding designing, planning, and policymaking, exclusive breastfeeding programmes and education about EBF practices should be considered. Moreover, it is also important to consider the management of EBF services, the design of the environment, and schedules that are conducive and friendly to working women with breastfeeding infants. To increase EBF prevalence rates, education about EBF practices should be emphasised, including education about the types of food that help increase milk production. Similarly, education can be the best tool to remove harmful traditional beliefs concerning EBF practices.

## Author Contributions

C.J.M. conceived the original idea, prepared the initial manuscript and approved the final draft of the paper.

## Funding

This research is free from any funding. The authors declare that no funds, grants, or other support were received during the preparation of this manuscript.

## Ethics Statement

No human participants have been used in this study.

## Conflicts of Interest

The authors declare no conflicts of interest.

## Transparency Statement

The lead author Chakupewa Joseph Mpambije affirms that this manuscript is an honest, accurate, and transparent account of the study being reported; that no important aspects of the study have been omitted; and that any discrepancies from the study as planned (and, if relevant, registered) have been explained.

## Data Availability

Data sharing is not applicable to this article as no new data were created or analysed in this study.

## References

[1] M. Wang and M. Ren , “World Health Day 2025: Time to Change Mindset Beyond Global Commitment to Maternal Health and Women's Well‐Being,” China CDC Weekly 7, no. 14 (2025): 449–452. 40376441 10.46234/ccdcw2025.074 PMC12075451

[2] World Health Organization . “Trends in maternal mortality 2000 to 2020: estimates by WHO, UNICEF, UNFPA, World Bank Group and UNDESA/Population Division”. World Health Organization (2023).

[3] Z. J. Ward , R. Atun , G. King , B. Sequeira Dmello , and S. J. Goldie , “Simulation‐Based Estimates and Projections of Global, Regional and Country‐Level Maternal Mortality by Cause, 1990–2050,” Nature Medicine 29, no. 5 (2023): 1253–1261. 10.1038/s41591-023-02310-x PMC10202807 37081226

[4] S. A. Mwijalilege , M. L. Kadigi , and C. Kibiki , “Comparing ARFIMA and ARIMA Models in Forecasting Under Five Mortality Rate in Tanzania,” Asian Journal of Probability and Statistics 27, no. 1 (2025): 107–121.

[5] I. Solarin , C. Dumbura , D. P. Lakhoo , et al., “Characteristics of Longitudinal Maternal Health Studies in Sub‐Saharan Africa: A Systematic Mapping of Literature Between 2012 and 2022,” International Journal of Gynaecology and Obstetrics: The Official Organ of the International Federation of Gynaecology and Obstetrics 169, no. 1 (2025): 51–62. 39548805 10.1002/ijgo.16035 PMC11911973

[6] L. Benova , M. Siddiqi , I. O. O. Abejirinde , and O. Badejo , “Time Trends and Determinants of Breastfeeding Practices Among Adolescents and Young Women in Nigeria, 2003–2018,” BMJ Global Health 5 (2020): e002516, 10.1136/bmjgh-2020-002516. PMC7412589 32764127

[7] C. Joseph and S. O. Maluka , “The Influence of Community Factors in the Implementation of Community‐Based Interventions to Improve Antenatal Care: A Qualitative Study Based on the Imcha Programme in Tanzania,” Reproductive Health 18 (2021): 188. 34551794 10.1186/s12978-021-01225-5 PMC8456547

[8] S. O. Maluka , C. J. Mpambije , P. C. Kamuzora , and S. Fitzgerald , “The Effects of Community‐Based Interventions on the Uptake of Selected Maternal and Child Health Services: Experiences of the IMCHA Project in Iringa Tanzania, 2015‐2020,” BMC Pregnancy and Childbirth 23 (2023): 328, 10.1186/s12884-023-05638-x. 37158851 PMC10165785

[9] WHO . Infant and young feeding [WWW Document]. Fact Sheet, https://www.who.int/en/news‐room/fact‐sheets/detail/infant‐and‐young‐child‐feeding. Global Breastfeeding Collective, WHO, UNICEF, 2019. Global Breastfeeding Scorecard 2019, Global Breastfeeding Scorecard. (2020) New York, Geneva.

[10] S. Wang , T. Zhang , K. Wang , D. Li , and X. Cao , “The Global Burden of Childhood Diarrheal Diseases Attributable to Suboptimal Breastfeeding From 1990 to 2021: An Exploratory Analysis of Estimates From the Global Burden of Disease Study,” International Breastfeeding Journal 20, no. 1 (2025b): 19. 40140930 10.1186/s13006-025-00713-9 PMC11948792

[11] A. R. Maonga , M. J. Mahande , D. J. Damian , and S. E. Msuya , “Factors Affecting Exclusive Breastfeeding Among Women in Muheza District Tanga Northeastern Tanzania: A Mixed Method Community Based Study,” Maternal and Child Health Journal 20 (2016): 77–87, 10.1007/s10995-015-1805-z. 26239611 PMC4712214

[12] WHO . “Malnutrition”, https://www.who.int/news‐room/fact‐sheets/detail/malnutriti, (2020).

[13] H. J. T. Mongboa , B. O. B. Mejiozem , and J. C. Gody , “Mothers' Knowledge and Practices Regarding Exclusive Breastfeeding in the Central African Republic,” Open Journal of Pediatrics 15 (2025): 74–92, 10.4236/ojped.2025.151008.

[14] A. M. Haughton and I. A. Michaelis , “Feeding Practices of Very Low Birth Weight Infants Born 2018 at a Tertiary Hospital in South Africa,” South African Journal of Clinical Nutrition 38 (2025): 48–54.

[15] H. M. Hassen , “Trends and Determinants of Exclusive and Predominant Breastfeeding Practices for Two Decades (2000–2019) in Ethiopia,” Frontiers in Nutrition 12 (2025): 1516547, 10.3389/fnut.2025.1516547. 39911801 PMC11794101

[16] Demographic and Health Survey and Malaria Indicator Survey . 2022. The United Republic of Tanzania.

[17] Ministry of Health (MoH) . [Tanzania Mainland], Ministry of Health (MoH) [Zanzibar], National Bureau of Statistics (NBS), Office of the Chief Government Statistician (OCGS), and ICF. Tanzania Demographic and Health Survey and Malaria Indicator Survey 2022 Final Report. Dodoma, Tanzania, and Rockville, Maryland, USA: MoH, NBS, OCGS, and ICF, 2022.

[18] T. H. Hussein , M. Mgongo , J. Katanga , et al., “Exclusive Breastfeeding Rates and Factors Associated With Exclusive Breastfeeding Practices in Northern Tanzania: Measurement Using Two Different Methodologies 24 Hours Recall and Recall Since Birth,” International Journal of Maternal and Child Health and AIDS (IJMA) 8, no. Issue 1 (2019): 32–43, 10.21106/ijma.258. 31049262 PMC6487506

[19] V. Mogre , M. Dery , and P. K. Gaa , “Knowledge, Attitudes and Determinants of Exclusive Breastfeeding Practice Among Ghanaian Rural Lactating Mothers,” International Breastfeeding Journal 11 (2016): 12. 27190546 10.1186/s13006-016-0071-z PMC4869336

[20] T. D. Kolawole , A. H. Okijiola , B. M. Ayodeji , E. J. Oluwafemi , O. E. Adebola , and B. Roseline , “Determinants of Exclusive Breastfeeding Practices Among Nursing Mothers at State Hospital, Ijaiye, Abeokuta, Ogun State,” Eurasian Journal of Human Health and Disease 1, no. 1 (2025): 15–25.

[21] M. W. Muluneh , “Determinants of Exclusive Breastfeeding Practices Among Mothers in Ethiopia,” PLoS One 18, no. 2 (2023): e0281576, 10.1371/journal.pone.0281576. 36758057 PMC9910689

[22] S. A. Ali , A. A. Dero , and S. A. Ali , “Factors Affecting the Utilization of Antenatal Care Among Pregnant Women: A Literature Review,” Journal of Pregnancy and Neonatal Medicine 2, no. 2 (2018): 41–45, https://www.researchgate.net/publication/328043592.

[23] D. V. Parums , “Editorial: Review Articles, Systematic Reviews, Meta‐Analysis, and the Updated Preferred Reporting Items for Systematic Reviews and Meta‐Analyses (PRISMA) 2020 Guidelines,” Medical Science Monitor 27 (2021): e934475, 10.12659/MSM.93. 34421116 PMC8394590

[24] C. Uk. Qualitative Research: Appraisal Tool. 10 Questions to Help You Make Sense of Qualitative Research [internet]. Critical Appraisal Skills Programme (CASP); (2018)2013.

[25] M. Kazaura , “Exclusive Breastfeeding Practices in the Coast Region, Tanzania,” African Health Sciences 16, no. Issue 1 (2016) School of Public Health, Epi/Biostat. 10.4314/ahs.v16i1.6 PMC4915437 27358612

[26] M. H. Rasheed , R. Philemon , G. D. Kinabo , M. Maxym , A. M. Shayo , and B. T. Mmbaga . “Adherence to Exclusive Breastfeeding and Associated Factors in Mothers of HIV‐Exposed Infants Receiving Care at Kilimanjaro Christian Medical Centre, Tanzania”. East African Health Research Journal; Volume 2, Number 1. (2018), www.eahealth.org. 10.24248/EAHRJ-D-16-00365 PMC8279206 34308173

[27] M. J. Mahande , D. Jeremia , and S. Msuya., “Factors Affecting Exclusive Breastfeeding Among Women in Muheza District Tanga Northeastern Tanzania: A Mixed Method Community Based Study,” Maternal and Child Health Journal 20, no. 1 (2015): 77–87. 10.1007/s10995-015-1805-z PMC4712214 26239611

[28] S. Grounds . Views of Breastfeeding in Public among Informally‐Working Mothers of Infants under 6 Months in Moshi Urban District, Kilimanjaro Region, Tanzania: A Qualitative Study. University of North Carolina at Chapel Hill. (2021) A Senior Honor Thesis.

[29] M. Mgongo , T. H. Hussein , B. Stray‐Pedersen , S. Vangen , S. E. Msuya , and M. Wandel , “Facilitators and Barriers to Breastfeeding and Exclusive Breastfeeding in Kilimanjaro Region, Tanzania: A Qualitative Study,” International Journal of Pediatrics 2019 (2019): 1–7, 10.1155/2019/8651010. PMC6378044 30853994

[30] G. Augustino , A. Anaeli , and B. F. Sunguya , “Barriers to Exclusive Breastfeeding Practice Among HIV‐Positive Mothers in Tanzania: An Exploratory Qualitative Study,” PLoS One 19, no. 5 (2024): e0296593, 10.1371/journal.pone.0296593. 38805480 PMC11132465

[31] C. R. Matare , H. C. Craig , S. L. Martin , et al., “Barriers and Opportunities for Improved Exclusive Breastfeeding Practices in Tanzania: Household Trials With Mothers and Fathers,” Food and Nutrition Bulletin 40, no. 3 (2019): 308–325, 10.1177/0379572119841961. 31067996 PMC7751330

[32] G. Augustino , A. Anaeli , and B. F. Sunguya , “Barriers to Exclusive Breastfeeding Practice Among HIV‐Positive Mothers in Tanzania. An Exploratory Qualitative Study,” Plos one 28 (2024): 1–18, 10.1371/journal.pone.029659328. PMC11132465 38805480

[33] M. Mgongo , T. H. Hussein , B. Stray‐Pedersen , S. Vangen , S. E. Msuya , and M. Wandel , “We Give Water or Porridge, but We Don't Really Know What the Child Wants:” a Qualitative Study on Women's Perceptions and Practises Regarding Exclusive Breastfeeding in Kilimanjaro Region, Tanzania,” BMC Pregnancy and Childbirth 18, no. 2018 (2018): 323, 10.1186/s12884-018-1962-3. 30089449 PMC6083497

[34] T. H. Hashim , M. Mgongo , J. Katanga , et al., “Predictors of Appropriate Breastfeeding Knowledge Among Pregnant Women in Moshi Urban, Tanzania: A Cross‐Sectional Study,” International Breastfeeding Journal 12, no. 1 (2017): 11. 28228840 10.1186/s13006-017-0102-4 PMC5307776

[35] K. S. Dede and H. Bras , “Exclusive Breastfeeding Patterns in Tanzania: Do Individual, Household, or Community Factors Matter?,” International Breastfeeding Journal 15, no. 1 (2020) (2020): 32. 32321557 10.1186/s13006-020-00279-8 PMC7178598

[36] H. C. Craig , C. R. Matare , S. L. Martin , et al., “Because of Mchango, I Give My Baby Gripe Water so He Sleeps and Stops Crying”: Exclusive Breastfeeding and Parents' Concerns about Colic‐Like Symptoms in Infants under 6 Months in Lake Zone, Tanzania,” World Nutrition 14, no. 3 (2023): 48–59.

[37] E. Eliufoo , S. Mgeyekwa , V. Majengo , T. Yusheng , and L. Yamin , “Exclusive Breastfeeding Experience Among Healthcare Working Mothers in Central Tanzania: A Qualitative Study,” Nursing Practice Today 11, no. 3 (2024): 259–271.

[38] K. Mwantimwa and N. Mwaisela , “Demographic Determinants of Access to and Usage of Breastfeeding Information Among Parents in Mbeya City, Tanzania,” University of Dar es Salaam Library Journal 13, no. 1 (2018): 20–35.

[39] E. Kokushubira , A. Kiwanuka , and S. Maluka , “Factors Affecting Exclusive Breastfeeding Among Postnatal Mothers in Kinondoni Municipality, Dar es Salaam,” International Journal of Public Health Research 5, no. 4 (2017): 42–48.

[40] O. F. Jahanpour , E. L. Okango , J. Todd , H. Mwambi , and M. J. Mahande , “Role of Clusters in Exclusive Breastfeeding Practices in Tanzania: A Secondary Analysis Study Using Demographic and Health Survey Data (2015/2016),” Frontiers in Pediatrics 10, no. 2022 (2022): 939706. 36263150 10.3389/fped.2022.939706 PMC9574076

[41] Z. Mchome , S. Yousefzadeh , A. Bailey , and H. Haisma , “When I Breastfeed, It Feels as If My Soul Leaves the Body”: Maternal Capabilities for Healthy Child Growth in Rural South‐Eastern Tanzania,” International Journal of Environmental Research and Public Health 17, no. 17 (2020) (2020): 6215. 32867111 10.3390/ijerph17176215 PMC7504657

[42] J. de Bruyn , B. Bagnol , I. Darnton‐Hill , W. Maulaga , P. C. Thomson , and R. Alders , “Characterizing Infant and Young Child Feeding Practices and the Consumption of Poultry Products in Rural Tanzania: A Mixed Methods Approach,” Maternal & Child Nutrition 14, no. 2 (2018) (2018): e12550. 29098763 10.1111/mcn.12550 PMC6866118

[43] K. S. S. Dede , P. E. Mosha , and G. A. Mgaza , “Determinants of Infants Feeding Practices in Tanzania: A Cross‐Sectional Analysis Among Breastfeeding Mothers in Masasi District, Tanzania,” Tengeru Community Development Journal 6, no. 2 (2019), www.ticd.ac.tz.

[44] H. Y. Lyellu , T. H. Hussein , M. Wandel , B. Stray‐Pedersen , M. Mgongo , and S. E. Msuya , “Prevalence and Factors Associated With Early Initiation of Breastfeeding Among Women in Moshi Municipal, Northern Tanzania,” BMC Pregnancy and Childbirth 20, no. 1 (2020) (2020): 285. 32393191 10.1186/s12884-020-02966-0 PMC7216396

[45] O. F. Jahanpour , E. L. Okango , J. Todd , H. Mwambi , and M. J. Mahande , “Mapping Regional Variability of Exclusive Breastfeeding and Its Determinants at Different Infant's Age in Tanzania,” BMC Pregnancy and Childbirth 23, no. 1 (2023): 769. 37924009 10.1186/s12884-023-06076-5 PMC10623860

[46] F. Mandara , C. Festo , E. Killel , et al., “The Relationship Between Feeding Practices and Stunting Among Children Under Two Years in Tanzania Mainland: A Mixed‐Method Approach,” Bulletin of the National Research Centre 48, no. 1 (2024) (2024): 112.

[47] M. C. Marais . Exploring the Factors Influencing Exclusive Breastfeeding Within the First 14 Weeks Postpartum With Mothers in the Khayelitsha‐Eastern Substructure (2020).

[48] S. R. Quebu , D. Murray , and U. B. Okafor , “Barriers to Exclusive Breastfeeding for Mothers in Tswelopele Municipality, Free State Province, South Africa: A Qualitative Study,” Children 10, no. 8 (2023): 1380. 37628379 10.3390/children10081380 PMC10453665

[49] N. A. Jama , A. Wilford , Z. Masango , et al., “Enablers and Barriers to Success Among Mothers Planning to Exclusively Breastfeed for Six Months: A Qualitative Prospective Cohort Study in Kwazulu‐Natal, South Africa,” International Breastfeeding Journal 12, no. 1 (2017) (2017): 43. 29026431 10.1186/s13006-017-0135-8 PMC5627494

[50] M. P. Hmone , M. J. Dibley , M. Li , and A. Alam , “A Formative Study to Inform Mhealth Based Randomized Controlled Trial Intervention to Promote Exclusive Breastfeeding Practices in Myanmar: Incorporating Qualitative Study Findings,” BMC Medical Informatics and Decision Making 16, no. 1 (2016): 60. 27260252 10.1186/s12911-016-0301-8 PMC4893226

[51] E. S. Seabela , P. Modjadji , and K. E. Mokwena , “Facilitators and Barriers Associated With Breastfeeding Among Mothers Attending Primary Healthcare Facilities in Mpumalanga, South Africa,” Frontiers in Nutrition 10 (2023): 1062817. 36998907 10.3389/fnut.2023.1062817 PMC10043338

[52] M. M. Thet , E. E. Khaing , N. Diamond‐Smith , M. Sudhinaraset , S. Oo T., and T. Aung., “Barriers to Exclusive Breastfeeding in the Ayeyarwaddy Region in Myanmar: Qualitative Findings From Mothers, Grandmothers, and Husbands,” Appetite 96 (2016): 62–69. 26344810 10.1016/j.appet.2015.08.044

[53] H. Khatun , C. A. Comins , R. Shah , M. Munirul Islam , N. Choudhury , and T. Ahmed , “Uncovering the Barriers to Exclusive Breastfeeding for Mothers Living in Dhaka's Slums: A Mixed Method Study,” International Breastfeeding Journal 13, no. 1 (2018): 44. 30275873 10.1186/s13006-018-0186-5 PMC6158891

[54] L. M. Andrew , “Prevalence and Factors Hindering First Time Mothers From Exclusively Breastfeeding in Kyabugimbi Health Center Iv, Bushenyi District Uganda,” (master's thesis, Kampala International University, 2017).

[55] J. Rujumba , G. Ndeezi , V. Nankabirwa , et al., “If I Have Money, I Cannot Allow My Baby to Breastfeed Only …” Barriers and Facilitators to Scale‐Up of Peer Counselling for Exclusive Breastfeeding in Uganda,” International Breastfeeding Journal 15, no. 1 (2020): 43. 32414404 10.1186/s13006-020-00287-8 PMC7229593

[56] A. S. Rahadian and Y. Astuti , “The Socio‐Cultural Context of Barriers to Exclusive Breastfeeding Practices Among Mothers in Karanganyar District Central Java Province,” Jurnal Promkes 11 (2023): 52–62.

[57] S. Wataka , P. Tumukunde , E. Kawala , R. Nekaka , and J. Nteziyaremye , “Exclusive Breastfeeding in Manafwa District, Eastern Uganda ‐ Opportunities and Challenges: A Mixed Method Community‐Based Study,” Prim Health Care 11, no. 1 (2021): 1–9.

[58] Z. Zhu , A. Narayan , S. Zhang , et al., “How the Marketing Practices of Commercial Milk Formula Companies Impact Infant Breastfeeding Practices in China,” BMJ Global Health 8, no. 11 (2023): e012803. 10.1136/bmjgh-2023-012803 PMC10649769 37949499

[59] C. Nsiah‐Asamoah , D. T. Doku , and S. Agblorti , “Mothers' and Grandmothers' Misconceptions and Socio‐Cultural Factors as Barriers to Exclusive Breastfeeding: A Qualitative Study Involving Health Workers in Two Rural Districts of Ghana,” PLoS One 15, no. 9 (2020) (2020): 0239278. 10.1371/journal.pone.0239278 PMC7498105 32941500

